# National Surgical, Obstetric, and Anesthesia Plans Supporting the Vision of Universal Health Coverage

**DOI:** 10.9745/GHSP-D-19-00314

**Published:** 2020-03-30

**Authors:** Alexander W. Peters, Lina Roa, Emile Rwamasirabo, Emmanuel Ameh, Mpoki M. Ulisubisya, Lubna Samad, Emmanuel M. Makasa, John G. Meara

**Affiliations:** a Department of Surgery, Weill Cornell Medical College, New York, NY, USA.; b Program in Global Surgery and Social Change, Department of Global Health and Social Medicine, Harvard Medical School, Boston, MA, USA.; c Department of Plastic and Oral Surgery, Boston Children's Hospital, Boston, MA, USA.; d Department of Obstetrics and Gynecology, University of Alberta, Edmonton, Alberta, Canada.; e Department of Surgery, King Faisal Hospital, Kigali, Rwanda.; f Department of Surgery, National Hospital, Abuja, Nigeria.; g Ministry of Health Community Development Gender Elderly and Children, Dar es Salaam, Tanzania.; h Center for Essential Surgical and Acute Care, Indus Health Network, Karachi, Pakistan.; i Public Service Management Division Cabinet Office, Office of the President, Lusaka, Zambia.; j Wits Centre of Surgical Care for Primary Health & Sustainable Development, School of Medicine, Faculty of Health Sciences, University of Witwatersrand, Johannesburg, South Africa.

## Abstract

Developing a national surgical, obstetric, and anesthesia plan is an important first step for countries to strengthen their surgical systems and improve surgical care. Barriers to successful implementation of these plans include data collection, scalability, and financing, yet surgical system strengthening efforts are gaining momentum in achieving universal access to emergency and essential surgical care.

## BACKGROUND

In 2018, 40 years after the Declaration of Alma-Ata, the global community renewed its commitment to universal health coverage (UHC) and reemphasized the importance of primary health care in health systems strengthening (HSS) in the Declaration of Astana.^1^^,^^2^ If we are to achieve UHC, several important synergistic health services must be considered beyond only primary health care. In particular, access to surgical, anesthesia, and obstetric services is essential to enhancing physical, mental, and social well-being.

Despite calls for surgery and health for all from World Health Organization (WHO) leaders as far back as 1980,^3^ surgical, obstetric, and anesthesia (SOA) care has often been deemed too expensive and complex to deliver at scale^4^ and has, until recently, remained largely neglected in global surgery policy.^5^ To address this gap, a surge of research, advocacy, policy, and implementation efforts to expand access to these services have been taking root in several low- and middle-income countries.

In 2015, the *Lancet* Commission on Global Surgery published a report that approximately 70% of the world still lacked access to safe, affordable emergency and essential SOA care when needed—a shortfall disproportionately affecting those living in low- and middle-income countries. The Commission proposed 6 indicators to measure surgical systems (Table 1).^4^^,^^6^^–^^8^

**TABLE 1.** *Lancet* Commission on Global Surgery Core Key Performance Indicators for Monitoring Surgical Systems

Indicator	*Lancet* Commission on Global Surgery Definition	*Lancet* Commission on Global Surgery Target by 2030	Included in the World Bank Group's World Development Indicators	Included in the World Bank Group's 2018 Atlas of Sustainable Development Goals	Included in the World Health Organization's Core 100 Indicators
Access to timely essential surgery	Proportion of the population that can access a facility within 2 hours that can do cesarean delivery, laparotomy, and open fracture repairs	A minimum of 80% coverage of essential surgical and anesthesia services per country	-	-	Yes
Specialist surgical workforce density	Number of specialist surgical, anesthetic, and obstetric physicians working, per 100,000 population	100% of countries with at least 20 surgical, anesthetic, and obstetric physicians per 100,000 population	Yes	Yes	Yes
Number of surgical procedures performed	Procedures done in an operating theatre, per 100,000 population per year	80% of countries by 2020 and 100% of countries by 2030 tracking surgical volume; a minimum of 5,000 procedures per 100,000 population	Yes	-	Yes
Perioperative mortality rate	All-cause death rate before discharge in patients who have undergone a procedure in an operating theater, divided by the total number of procedures	80% of countries by 2020 and 100% of countries by 2030 tracking perioperative mortality; in 2020, assess global data and set national targets for 2030	-	-	Yes
Protection against impoverishing expenditure for surgical care	Proportion of households protected against impoverishment from direct out-of-pocket payments for surgical and anesthesia care	100% protection against impoverishment from out-of-pocket payments for surgical and anesthesia care	Yes	-	Yes
Protection against catastrophic expenditure for surgical care	Proportion of households protected against catastrophic expenditure from direct out-of-pocket payments for surgical and anesthesia care	100% protection against catastrophic expenditure from out-of-pocket payments for surgical and anesthesia care	Yes	-	Yes

In the same year, the World Health Assembly passed a resolution (WHA 68.15) that declared emergency and essential surgical and anesthesia care as essential components of UHC,^9^ and the World Bank Group described 44 cost-effective surgical interventions in *Disease Control Priorities*.^10^ SOA care has become increasingly viewed as integral to achieving the United Nations Sustainable Development Goals,^11^ and global leaders have begun to call for greater investments in surgical care.^12^

This article reviews the health policy roadmaps that 5 countries have developed since WHA 68.15 as they strive to include equitable access to SOA care in their health programs.

## NATIONAL SURGICAL, OBSTETRICS, AND ANESTHESIA PLAN FRAMEWORK

Since 2015, several countries in Africa and Asia have begun to integrate national surgical, obstetric, and anesthesia plans (NSOAPs) into their country's national health strategic plans. These innovative, context-specific NSOAPs are meant to fit within the country's broader HSS initiatives. Their ultimate goal is to guide countries or regions to identify and close gaps as they move toward achieving, by 2030, the core surgical benchmarks necessary to deliver UHC and fulfill their commitments to WHA 68.15.

**FIGURE 1:**
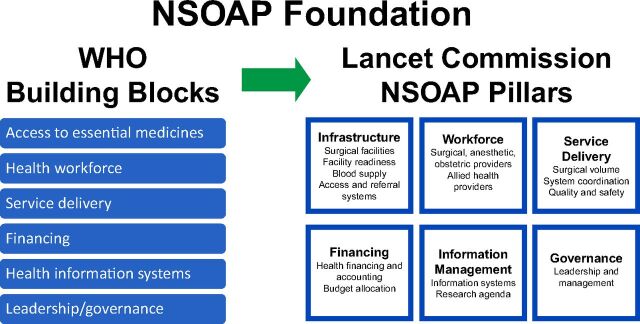
Adapting WHO Building Blocks for NSOAPs^14^ Abbreviations: NSOAP,National Surgical, Obstetric, and Anesthesia Plan; WHO, World Health Organization.

The NSOAP development process is founded on 6 core domains adapted from the World Health Organization (WHO) HSS Building Blocks.^13^ The process replaces access to essential medicines with surgical infrastructure and places the medicines required for surgical care (e.g., anesthetic agents) within care delivery itself (Figure 1).^14^ These design parallels have allowed NSOAP development processes to complement other HSS initiatives and fit within broader WHO policy frameworks.

**FIGURE 2:**
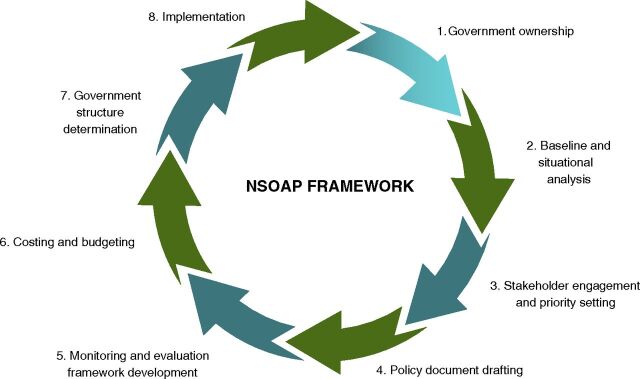
NSOAP Creation Theoretical Framework (Adapted^11^^–^^13^) Abbreviation: NSOAP, National Surgical, Obstetric, and Anesthesia Plan.

**FIGURE 3:**
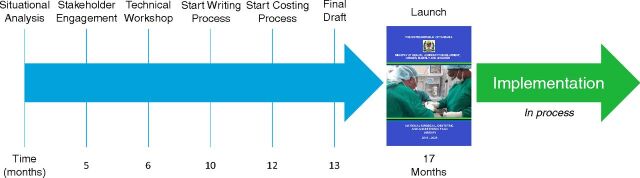
Tanzania NSOAP Development Process Timeline Abbreviation: NSOAP, National Surgical, Obstetric, and Anesthesia Plan.

To facilitate adoption, the development process follows a flexible 8-step theoretical framework (Figure 2) to drive health policy reforms that will improve access to surgical care while engaging all key stakeholders.^15^^–^^17^ This framework begins from within or by obtaining support from the ministry of health and follows specific steps that allow for baseline assessments, stakeholder engagement, policy formulation, monitoring and evaluation, costing, governance, and implementation.^15^ In Tanzania, where NSOAP implementation is underway, the planning process took approximately 17 months (Figure 3). The core output of the NSOAP framework is a context-specific, baselined, costed, consensus plan with clear monitoring, evaluation, and governance for investing in and implementing surgical scale-up in any province, country, or region.

## NOTABLE NSOAP DEVELOPMENT PROCESSES

Around the world, countries have started developing NSOAPs to sustainably expand access to SOA care. We describe 5 examples with which the authors have been closely involved and that demonstrate country-led processes that have focused on addressing health system gaps, integrating surgical policies into broader national policies, and including and consulting all relevant stakeholders in the planning process. However, it must be noted that this list is not exhaustive. Important country and regional-level efforts are taking place worldwide, including in Ethiopia, Madagascar, and Vietnam.

### Zambia

The Republic of Zambia has led efforts to expand access to surgical care, sponsoring and chairing the diplomatic negotiations that culminated in the adoption of WHA68.15 in 2015 as well the follow-up Resolution WHA70(22) in 2017 that requires WHO member states to report on their progress of WHA68.15 every 2 years.^18^

The Zambian Ministry of Health brought together many different surgical system stakeholders to adopt the NSOAP. Two prior nationwide assessments, the emergency obstetrics and newborn care survey, and the emergency and essential surgical care capacity survey provided the baseline from which the Zambian NSOAP was structured. The fully costed Zambian NSOAP (2017–2021)^19^ that was launched at the World Health Assembly in Geneva in 2016 marked the first NSOAP modeled on the *Lancet* Commission's theoretical framework and the first country to affirm its political commitment to WHA 68.15.^18^ The NSOAP has since been fully integrated into the Zambian National Health Strategic Plan 2017–2021,^20^ which is a key part of Zambia's broader National Development Plan.^21^ Through this integrated process, the Republic of Zambia served as a model for incorporating NSOAP implementation within a country's broader sustainable development agenda. Furthermore, Zambia has also sponsored and chaired the adoption of regional resolutions aimed at closing the gaps in SOA care at the East Central and Southern African Health Community in 2017 and the Southern African Development Community (SADC) Health Ministers Conferences in Windhoek in 2018^22^ and Dar es Salaam in 2019.^23^

Through the NSOAP process, the Republic of Zambia served as a model of strong national ownership, advocacy, leadership, and coordination.

### Tanzania

The Tanzanian NSOAP development process, completed in 2018, sought to include a wide range of stakeholders at every step of policy dialogue and creation.^24^ Stakeholders were engaged in 4 key phases, and over 200 diverse stakeholders were interviewed through a situational analysis to better understand local challenges to providing quality surgical care. To accomplish this, the NSOAP development team visited health facilities at each level of care delivery and interviewed frontline providers, training institutions, blood banks, health insurers, government officials and private practitioners. This allowed them to obtain a comprehensive picture of the gaps in surgical care that could inform their priority setting.^25^ During the priority-setting phase, more than 70 stakeholders were engaged in policy dialogue to establish priority areas of the plan based on the situation analysis and their on-the-ground experiences. The plan was then drafted, costed, and ultimately adopted and signed by the Tanzanian Ministry of Health in 2018.

As a result of this bottom-up approach, the Tanzanian NSOAP reflects the challenges facing all actors in the surgical ecosystem, including frontline providers who will ultimately implement the plan, ensuring their buy-in and ownership from the beginning.

Based on the results of a situational analysis involving key stakeholders, the Tanzanian NSOAP reflects the challenges facing all actors in the surgical ecosystem.

### Pakistan

**FIGURE 4:**
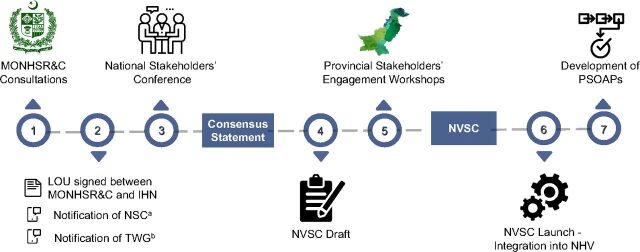
Roadmap for Pakistan's Surgical Care Strengthening, From National Vision to Provincial Plans Abbreviations: IHN, Indus Health Network; LOU, letter of understanding; MONHSR&C, Ministry of National Health Services, Regulation, and Coordination; NHV, National Health Vision; NSC, National Steering Committee; NVSC, National Vision for Surgical Care; PSOAP, Provincial, Surgical, Obstetric, and Anesthesia Plan; TWG, technical working group. ^a^ Consisting of representatives from international and national public and private stakeholders to oversee and coordinate the process of being the decision maker. ^b^ Consisting of international and national partners to conduct research, provide technical support throughout the process, and draft documents.

The NSOAP theoretical framework provides a flexibility that can also be applied in a decentralized, context-specific manner. In Pakistan, due to its large population, the federal government regulates and coordinates the overall strategic approach to health care provision. However, priority setting, policy, and service implementation is devolved to provincial governments. The forthcoming Pakistani *National Vision for Surgical Care 2025*, a context-specific NSOAP development process begun in November 2018, will establish a guiding vision for surgery that aligns with Pakistan's federal-level *National Health Vision 2016–2025* (Figure 4). Pakistan's NSOAP aims specifically to include children's surgery to more effectively serve the one-third of its' population under the age of 15.^26^ It also provides a roadmap for each individual provincial government to identify local barriers to surgical care, develop individually tailored provincial SOAPs, and implement context-specific changes within their provincial health networks.^27^ This unique approach specifically tailored to the Pakistani context provides a model for other countries with regional health authorities to adapt the NSOAP framework to their particular governance structures.

### Rwanda

In 2017, the Rwandan Ministry of Health, together with Rwandan professional societies and international academic collaborators, embarked on a systematic baseline assessment of surgical care across the country. In a simultaneous effort to bolster surgical research capacity, this assessment was led by active Rwandan surgical residents in all of Rwanda's 42 district hospitals using modified WHO assessment tools to measure facility and health system preparedness for emergency and essential surgery services. Using the *Lancet* Commission's framework, data were organized and analyzed around the 6 surgical indicators and consequently 5 intervention domains were established. Surgical workforce (e.g., surgeons, anesthetists, obstetricians, nurses, etc.) was identified as the largest barrier to providing essential surgical care in Rwanda and became the main focus of the NSOAP development process.^28^

In Rwanda, surgical workforce—identified as the largest barrier to providing essential surgical care—became the focus of the NSOAP process.

Consensus on targets, strategies, activities, and financing was reached through 3 intensive workshops that brought together public and private stakeholders from every level. The Rwanda NSOAP was launched in December 2018, and then integrated into the Health Sector Strategic Plan 2018–2019 in January 2019.^28^ Since this launch, NSOAP monitoring tools have been embedded into Rwanda's health management information systems, and a steering committee has been designated to meet on a quarterly basis to monitor progress of surgical care across Rwanda.

### Nigeria

In 2017, Nigeria embarked on a national surgical, obstetric, anesthesia, and nursing plan (NSOANP) process that was driven first by the national surgical and anesthesia societies and then ultimately taken up by the Federal Ministry of Health. This stepwise engagement ensured provider buy-in from the beginning and will likely be central to the success of forthcoming NSOANP implementation. In Nigeria, the federal government creates health care policies and priorities through the Federal Ministry of Health. However, implementation of these policies is done both centrally through federally owned tertiary health institutions and the primary health care development agency as well as at the state level through separate local health authorities.

Strategic Priorities for Surgical Care (StraPS), Nigeria's NSOANP, created prioritized surgical system targets and an implementation roadmap that includes monitoring, evaluation, and feedback for central and state governments to follow. StraPS is unique for several reasons. In Nigeria, children under 15 years old constitute 43% of the population of 199 million. StraPS included children's surgery in a surgical plan for the first time and addressed the surgical needs of this unique demographic. In addition, StraPS specifically included nursing care, which forms an inseparable component of surgical quality and safety, to ensure that nursing is captured in surgical training and workforce development programs.

Nigeria prioritized children's surgery and nursing care in its NSOAP.

Similar to other surgical plans, StraPS was structured to be integrated into Nigeria's existing National Strategic Health Development Plan 2018–2022 rather than exist as a standalone vertically implemented health policy.^29^

## CURRENT CHALLENGES TO NSOAP PROGRESS

Developing an NSOAP is an important first step toward surgical system strengthening as it formalizes a country's intention to improve surgical care and charts a roadmap for addressing real gaps in the health system as ascertained through baseline assessments. Nonetheless, having a plan for surgical system reform does not guarantee that implementation of the plan or meaningful change will occur. Despite the early successes of NSOAP development, implementation and scalability still face several barriers. Overcoming the barriers to implementation in each country will take engagement and collaboration from a diverse group of national and international stakeholders.

### Financing for Implementation

The World Bank Group urged that access to essential surgery should be financed early on in any nation's path to UHC.^10^ To do this, ministries of health and finance should be involved from the earliest stages of NSOAP development to best advocate for NSOAP financing among competing priorities in health system budgeting. An important step toward guaranteeing a budget line for NSOAP implementation within a ministry of health's broader budget is by integrating an NSOAP into a country's national health strategic plan, as several countries have done. NSOAP advocates can combine data on the current state of a country's surgical system with cost-effectiveness analyses and estimates of the potential macroeconomic benefits of investing in surgical care to gain early political support for including surgical care in HSS. Clear economic incentives exist for those financing HSS to consider including investments in surgical scale-up; the *Lancet* Commission reported that investments in surgical scale-up across low- and middle-income countries totaling approximately $350 billion could avert gross domestic product losses of US$12 trillion.^4^

Ministries of health and finance should be involved from the earliest stages of NSOAP development to best advocate for financing among competing health system budgeting priorities.

In Tanzania, where the NSOAP development process was completed in 2018 (Figure 3), the total cost of implementation is estimated at US$600 million by 2025, or US$1.7 per capita per year.^24^ As health budgeting and spending varies in each of the 5 countries described in this article (Table 2),^30^ no single funding source is expected to back this goal. NSOAP leaders should engage both domestic and international sources of financing early on. Organizations such as the United States Agency for International Development, the Bill & Melinda Gates Foundation, and the World Bank's Global Financing Facility may have new opportunities to align their existing programmatic priorities around maternal and child health, especially those regarding obstetric care, and around neglected tropical diseases with overlapping NSOAP priorities, such as blood banking and infection control.^31^^–^^33^

**TABLE 2.** Overview of Health Spending, by Country, 2016^30^

	Health Spending per Capita, USD^a^	Health Spending per GDP, %	Government Health Spending per Total Health Spending, %	Out-of-Pocket Spending per Total Health Spending, %	Development Assistance for Health per Total Health Spending, %
Nigeria	71.0	2.4	14.5	75.2	8.6
Pakistan	41.0	2.7	26.2	62.7	8.3
Rwanda	44.0	5.0	37.0	8.1	43.6
Tanzania	41.0	4.0	34.3	22.8	41.6
Zambia	64.0	3.2	38.1	12.3	44.0

Abbreviations: GDP, gross domestic product; USD, United States dollar.

a In 2018 purchasing power parity US dollars.

In Zambia, where NSOAP implementation has been slow, a new strategy has focused on finding entry points into existing health programs so that financial resources can be synergistically leveraged. Implementation is now poised to start with a small pilot program to generate evidence that demonstrates the impact and cost-effectiveness of surgical services with the intention of applying lessons learned toward broader Zambian surgical system scale-up.

### Scalability and Regionalization

Further adoption and implementation of NSOAPs needs to be considered both within countries and in geographic regions to see broad improvements in surgical care globally. Programmatic implementation of NSOAPs as new health policies could be advanced through pilot programs within subnational states or provinces to evaluate their impact on HSS and health outcomes before nationwide scale-up of SOA services. Furthermore, despite an increasing number of countries developing and beginning to implement surgical health policies, integration of these policies into their broader national health strategic plans remains a challenge in some cases.

To facilitate scale-up among SADC nations, regional-level cooperation has been shown to provide support and guidance for countries newly embarking on the NSOAP development process. Across the SADC nations, health ministers reaffirmed their commitment to WHA 68.15 in 2018;^22^ in 2019, they agreed to support the acceleration and completion of NSOAPs by formulating a regional SOA strategy together with a regional monitoring and accountability framework.^23^

Similarly, 14 nations across the South Pacific came together to measure the 6 *Lancet* Commission surgical key performance indicators (Table 1) in collaboration with Australia and New Zealand.^34^ Exemplifying the impactful role that professional association of high-income countries can have when aligned with regional priorities, the Royal Australasian College of Surgeons has supported the broader region with data collection, workforce training, and overall surgical scale-up. In 2019, at the Pacific Health Ministers Meeting in French Polynesia, 22 ministers of health or their designates from Pacific Island Countries & Territories committed to developing NSOAPs as a tool for strengthening surgical care in the region.

Such regionalization also creates an opportunity for WHO regional offices to better engage with member states that are committed to strengthening surgical care. For example, at the 72^nd^ World Health Assembly in 2019, Dr. Takeshi Kasai, WHO Regional Director for the Western Pacific, committed to incorporating surgical systems strengthening into its regional health strategy. These regional and country-level offices can provide technical support to policymakers and catalyze financing for NSOAP development within the context of existing national health plans and broader regional priorities. They can also coordinate surgical care improvements within existing programs that are already working on emergency care, maternal and child health, noncommunicable diseases, and other overlapping health priorities. As the global momentum for UHC grows, regional offices can ensure that essential SOA care is included in UHC planning.

### Data Collection

Systematic and sustainable data collection remains an essential component of the NSOAP development process. Adequate baselining and needs assessments must guide health sector prioritization, and ongoing data collection will allow for effective monitoring and evaluation of surgical system strengthening. Despite these advantages, data collection around SOA care remains limited.

To improve data collection efforts, ministries of health must support robust monitoring and evaluation plans to promote accountability around health financing and measure the impact of health reforms. To facilitate data reporting to projects like the World Bank's World Development Indictors, surgical questions must be integrated into widely used data collection mechanisms (e.g., Demographic and Health Surveys); such efforts are already underway in Zambia.

To improve data collection efforts, ministries of health must support robust monitoring and evaluation plans to promote accountability around health financing and measure the impact of health reforms.

Furthermore, academic collaborations and international professional societies have played an essential role in building research capacity, data collection, and analysis.^34^^–^^38^ The international nature of medical professional societies has provided them with a unique opportunities to work with several stakeholders to support surgical system strengthening efforts. For example, the World Federation of Societies of Anesthesiology has mapped and tracked the global anesthesia workforce and has led training and advocacy initiatives to increase the skilled anesthesia workforce. They also developed International Standards for Safe Practice of Anesthesia^39^ as well as an Anesthesia Facility Assessment tool.^40^ The International Federation of Gynecology and Obstetrics has led capacity building, training, and guideline development efforts for fistula surgery, management of postpartum hemorrhage, and cesarean hysterectomy and also collaborates closely with the International Congress of Midwives. As nurses and midwives provide a significant amount of surgical-related care, their inclusion in NSOAP development is paramount.

## THE WAY FORWARD

Given that 5 billion people lack access to surgical care,^4^ NSOAPs that promote equitable access to safe surgical, obstetric, nursing and anesthesia services can play a critical step for improving access to essential health services worldwide. NSOAP development will complement national health plans and be an important step toward achieving UHC and the Sustainable Development Goals.^15^^,^^41^

Surgical system strengthening efforts continue to gain momentum with dedicated international forums,^12^ broad adoption at WHO's Emergency and Essential Surgical Care Programme,^42^^,^^43^ and stakeholder engagement meetings for surgical care in Africa, Asia, and Latin America all occurring more frequently.^44^ Embarking on the NSOAP development process is only a first step toward improving access to surgery. Widespread implementation and financing of surgical care will take time. These important first steps are inching us closer to universal access to emergency and essential surgical care—and UHC—for all.

## Notes

**Peer Reviewed**

**First published online:** March 20, 2020

## References

[1] World Health Organization (WHO), United Nations Children's Fund (UNICEF). Primary Health Care: Report of the International Conference on Primary Health Care, Alma Ata, USSR, 6–12 September 1978. WHO and UNICEF; 1978. https://apps.who.int/iris/handle/10665/39228.

[2] World Health Organization (WHO); United Nations Children's Fund (UNICEF). Declaration of Astana, the Global Conference on Primary Health Care. Astana: WHO, UNICEF; 2018. https://www.who.int/docs/default-source/primary-health/declaration/gcphc-declaration.pdf. Accessed March 7, 2019.

[3] Mahler H. Surgery and Health for All: Address by Dr. H. Mahler Director-General of the World Health Organization to the XXII Biennial World Congress of the International College of Surgeons. 1980.

[4] Meara JG Leather AJM Hagander L. Global Surgery 2030: evidence and solutions for achieving health, welfare, and economic development. Lancet. 2015;386(9993):569–624. 10.1016/s0140-6736(15)60160-x. 25924834

[5] Farmer PE Kim JY. Surgery and global health: a view from beyond the OR. World J Surg. 2008;32(4):533–536. 10.1007/s00268-008-9525-9. 18311574 PMC2267857

[6] World Health Organization (WHO). 2018 Global Reference List of 100 Core Health Indicators (plus Health-Related SDGs). Geneva: WHO; 2018. https://apps.who.int/iris/bitstream/handle/10665/259951/WHO-HIS-IER-GPM-2018.1-eng.pdf?sequence=1. Accessed August 4, 2019.

[7] World Bank Group. Atlas of Sustainable Development Goals 2018: From World Development Indicators. New York: The World Bank; 2018. 10.1596/978-1-4648-1250-7

[8] The World Bank: Open Data (World Development Indicators), 2017. http://databank.worldbank.org/. Published 2017. Accessed May 5, 2019.

[9] World Health Organization (WHO). World Health Assembly Resolution 68.15: Strengthening Emergency and Essential Surgical Care and Anaesthesia as a Component of Universal Health Coverage. Geneva: WHO; 2015. http://apps.who.int/gb/ebwha/pdf_files/WHA68/A68_R15-en.pdf. 10.1007/s00268-015-3153-y 26239773

[10] Debas HT Donkor P Gawande A Jamison DT Kruk ME Mock CN. Disease Control Priorities, Third Edition (Volume 1): Essential Surgery. New York: The World Bank; 2015. 10.1596/978-1-4648-0346-8 26740991

[11] Roa L Jumbam DT Makasa E Meara JG. Global surgery and the sustainable development goals. Br J Surg. 2019;106(2):e44–e52. 10.1002/bjs.11044. 30620060

[12] Ghebreyesus TA. Video Address to the National Surgical, Obstetric, and Anesthesia Planning Conference for High-level Global, Regional and Country Authorities and Funders. 2019. https://www.pgssc.org/2019-national-surgical-planning

[13] World Health Organization (WHO). Everybody's Business: Strengthening Health Systems to Improve Health Outcomes, WHO's Framework for Action. Geneva: WHO; 2007. https://www.who.int/healthsystems/strategy/everybodys_business.pdf. Accessed November 18, 2018.

[14] Meara JG Peters AW. Theoretical Framework of the NSOAP Process. Paper presented at: Pakistan National Vision for Surgical Care 2025 Stakeholders Conference, November 15–16, 2018.

[15] Albutt K Sonderman K Citron I. Healthcare Leaders Develop Strategies for Expanding National Surgical, Obstetric, and Anaesthesia Plans in WHO AFRO and EMRO Regions. World J Surg. 2019;43(2):360–367. 10.1007/s00268-018-4819-z. 30298283

[16] Albutt K Citron I Sonderman K. National Surgical Obstetric and Anaesthesia Planning: Process and Consensus Recommendations. Dubai: Harvard Medical School Center for Global Health Delivery–Dubai; 2018. https://www.pgssc.org/dubai-nsoap-workshop. Accessed July 27, 2018.

[17] Sonderman KA Citron I Mukhopadhyay S. Framework for developing a national surgical, obstetric and anaesthesia plan. BJS Open. 2019;3(5):722–732. 10.1002/bjs5.50190. 31592517 PMC6773655

[18] World Health Organization. World Health Assembly Resolution 70(22): Progress in the Implementation of the 2030 Agenda for Sustainable Development; 2017. http://apps.who.int/gb/ebwha/pdf_files/WHA70/A70(22)-en.pdf. Accessed September 1, 2019.

[19] Republic of Zambia Ministry of Health. National Surgical, Obstetric and Anesthesia Strategic Plan (NSOAP): Year 2017–2021. Republic of Zambia Ministry of Health; 2017. https://docs.wixstatic.com/ugd/d9a674_70f6813fe4e74c4d99eb028336a38745.pdf. Accessed November 18, 2018.

[20] Republic of Zambia Ministry of Health. Zambia National Health Strategic Plan 2017–2021. Lusaka: Republic of Zambia Ministry of Health; 2017. https://www.moh.gov.zm/docs/ZambiaNHSP.pdf. Accessed August 4, 2019.

[21] Ministry of National Development Planning. Seventh National Development Plan (7NDP) 2017–2021. Lusaka: Republic of Zambia Ministry of National Development Planning; 2017. https://www.mndp.gov.zm/wp-content/uploads/2018/05/7NDP.pdf. Accessed August 4, 2019.

[22] Southern African Development Community. Statement: SADC Ministers of Health and Ministers Responsible for HIV and AIDS Meet in Namibia. November 8, 2018. 2018.

[23] Southern African Development Community. Record in English: Joint Meeting of the SADC Ministers of Health and Ministers Responsible for HIV and AIDS. November 7, 2019. 2019.

[24] The United Republic of Tanzania Ministry of Health Community Development Gender Elderly and Children. National Surgical, Obstetric and Anesthesia Plan (NSOAP): 2018–2025; 2018. https://docs.wixstatic.com/ugd/d9a674_4daa353b73064f70ab6a53a96bb84ace.pdf. Accessed November 18, 2018.

[25] Citron I Jumbam D Dahm J. Towards equitable surgical systems: development and outcomes of a national surgical, obstetric and anaesthesia plan in Tanzania. BMJ Glob Heal. 2019;4(2):e001282. 10.1136/bmjgh-2018-001282. 31139445 PMC6509614

[26] Siddiqui S Vervoort D Peters AW. Closing the gap of children's surgery in Pakistan. World J Pediatr Surg. 2019;2(1):e000027. 10.1136/wjps-2018-000027

[27] Pakistani National Vision for Surgeon Care 2025 Stakeholders Conference, November 15–16, 2018. 2018.

[28] Republic of Rwanda. National Surgical, Obstetrics, and Anesthesia Plan 2018–2024. Kigali: Republic of Rwanda; 2018. http://www.moh.gov.rw/fileadmin/Publications/Strategic_Plan/NSOAP_Rwanda-_Approved1.pdf. Accessed August 4, 2019.

[29] Federal Government of Nigeria. Second National Strategic Health Development Plan, 2018–2022. Abuja: Government of Nigeria; 2018.

[30] Global Burden of Disease Health Financing Collaborator Network. Past, present, and future of global health financing: a review of development assistance, government, out-of-pocket, and other private spending on health for 195 countries, 1995–2050. Lancet. 2019;393(10187):2233–2260. 10.1016/s0140-6736(19)30841-4. 31030984 PMC6548764

[31] Peters AW Pyda J Menon G Suzuki E Meara JG. The World Bank Group: Innovative financing for health and opportunities for global surgery. Surgery. 2019;165(2):263–272. 10.1016/j.surg.2018.07.040. 30274731

[32] Sonderman KA Citron I Albutt K Salaam-Blyther T Romanzi L Meara JG. USAID: Current support for global surgery and implications of reform. Surgery. 2018;164(6):1147–1155. 10.1016/j.surg.2018.05.074. 30249431

[33] Koch R Roa L Pyda J Kerrigan M Barthélemy E Meara JG. The Bill & Melinda Gates Foundation: An opportunity to lead innovation in global surgery. Surgery. 2019;165(2):273–280. 10.1016/j.surg.2018.08.002. 30316576

[34] Guest GD McLeod E Perry WRG. Collecting data for global surgical indicators: a collaborative approach in the Pacific Region. BMJ Glob Heal. 2017;2(4):e000376. 10.1136/bmjgh-2017-000376. 29225948 PMC5717952

[35] Massenburg BB Saluja S Jenny HE. Assessing the Brazilian surgical system with six surgical indicators: a descriptive and modelling study. BMJ Glob Heal. 2017;2(2):e000226. 10.1136/bmjgh-2016-000226. 28589025 PMC5444087

[36] Holmer H Bekele A Hagander L. Evaluating the collection, comparability and findings of six global surgery indicators. Br J Surg. 2019;106(2):e138–e150. 10.1002/bjs.11061. 30570764 PMC6790969

[37] Anderson GA Ilcisin L Abesiga L. Surgical volume and postoperative mortality rate at a referral hospital in Western Uganda: Measuring the Lancet Commission on Global Surgery indicators in low-resource settings. Surgery. 2017;161(6):1710–1719. 10.1016/j.surg.2017.01.009. 28259351

[38] Sonderman KA Muhawenimana E Taylor K. Use of a baseline assessment of Rwanda's health system to inform a national surgical, obstetric, and anesthesia strategic plan. J Am Coll Surg. 2018;227(4):S134–S135. 10.1016/j.jamcollsurg.2018.07.273

[39] Gelb AW Morriss WW Johnson W Merry AF, Workgroup the IS for a SP of A. World Health Organization-World Federation of Societies of Anaesthesiologists (WHO-WFSA) International Standards for a Safe Practice of Anesthesia. Anesth Analg. 2018;126(6):2047–2055. 10.1213/ane.0000000000002927. 29734240

[40] World Federation of Societies of Anaesthesiologists. Anaesthesia Facility Assessment Tool. https://www.wfsahq.org/resources/anaesthesia-facility-assessment-tool. Accessed August 4, 2019.

[41] Sonderman KA Citron I Meara JG. National surgical, obstetric, and anesthesia planning in the context of global surgery: the way forward. JAMA Surg. 2018;153(10):959–960. 10.1001/jamasurg.2018.2440. 30090937

[42] World Health Organization (WHO). Emergency and essential surgical care. https://www.who.int/surgery/en/. Accessed March 7, 2019.

[43] Ibbotson GC Meara JG Johnson W. Surgery for All: Social Justice for All. UN Special. 2019. https://www.unspecial.org/2019/05/surgery-for-all-social-justice-for-all/.

[44] Roa L Peters AW Meara JG, eds. National Surgical, Obstetric and Anesthesia Planning for High-Level Global, Regional, and Country Authorities and Funders. Proceedings of the Harvard Medical School Center for Global Health Delivery–Dubai. In: Dubai, UAE: Harvard Medical School Center for Global Health Delivery–Dubai; 2019. https://ghd-dubai.hms.harvard.edu/files/ghd_dubai/files/nsoap-2019-1.pdf.

